# Incomplete Kawasaki Disease Complicated by Shock: A Diagnostic Challenge in a Child

**DOI:** 10.7759/cureus.104268

**Published:** 2026-02-25

**Authors:** Mohamad Sabsabee, Nur Sabsabee, Alaa Qanbar, Mohamed Kandath, Moza Alhammadi, Shamma Lootah, Elham Al Fakih

**Affiliations:** 1 Pediatrics, Al Jalila Children's Speciality Hospital, Dubai, ARE; 2 Pediatrics, University of Kalamoon, Kalamoon, SYR; 3 Infectious Diseases, Al Jalila Children's Speciality Hospital, Dubai, ARE; 4 Pediatric Rheumatology, Al Jalila Children's Speciality Hospital, Dubai, ARE

**Keywords:** cervical lymphadenitis, coronary artery involvement, intravenous immunoglobulin (ivig), kawasaki disease (kd), kawasaki disease shock syndrome

## Abstract

Kawasaki disease (KD) is an acute vasculitis of medium-sized vessels that primarily affects children. It can present with incomplete or atypical features, leading to diagnostic delay and increased risk of cardiovascular complications. Kawasaki disease shock syndrome (KDSS) is a rare but severe manifestation characterized by hemodynamic instability and heightened inflammatory response. We report the case of an eight-year-old previously healthy boy who presented with fever and unilateral cervical lymphadenitis following recent travel. Initial evaluation suggested an infectious etiology, and he was treated with broad-spectrum antibiotics without clinical improvement. KD was suspected, and he was treated with intravenous immunoglobulins (IVIG) despite not fulfilling the full criteria for KD. Subsequently, he developed a diffuse maculopapular rash, non-purulent conjunctivitis, strawberry tongue, and escalating inflammatory markers, increasing the suspicion of incomplete KD. Despite initial treatment with intravenous immunoglobulin (IVIG), the patient acutely deteriorated with hypotension and respiratory distress, requiring intensive care admission and inotropic support. Repeat echocardiography later demonstrated coronary artery dilation and reduced left ventricular systolic function, confirming the diagnosis of KD complicated by shock. He was successfully treated with a second dose of IVIG, high-dose aspirin, and systemic corticosteroids, with subsequent clinical and hemodynamic recovery. This case highlights the diagnostic challenges of incomplete KD, the need for heightened clinical suspicion in children with persistent fever and hyperinflammation, and the need for early recognition and aggressive management of Kawasaki shock syndrome to prevent cardiac complications.

## Introduction

Kawasaki disease (KD) is a self-limited systemic vasculitis of childhood that affects medium-sized arteries, with a particular predilection for the coronary arteries. In the developed countries, it remains the leading cause of acquired heart disease in children and a major cause of long-term cardiovascular morbidity when not promptly recognized and treated [1]. The incidence varies significantly by geographic region, with the highest rates reported in East Asia, particularly Japan, while lower but steadily increasing rates are observed in North America and Europe. KD predominantly affects children under five years of age, with a peak incidence between 6 months and 2 years, and shows a slight male predominance. Seasonal clustering has also been described, most commonly in winter and early spring, suggesting a possible infectious or environmental trigger [1-2]. The diagnosis of classic KD requires the fever to be present for at least 5 days in combination with characteristic clinical features, including polymorphous rash, bilateral non-purulent conjunctivitis, mucosal changes, extremity changes, and cervical lymphadenopathy [2].

Incomplete or atypical KD refers to cases in which children do not fulfill the full clinical criteria yet exhibit laboratory and echocardiographic features consistent with the disease. This form is particularly common in infants and older children and is associated with delayed diagnosis and a higher risk of coronary artery abnormalities [2,3]. Cervical lymphadenitis-dominant presentations are a recognized atypical phenotype and frequently lead to an initial misdiagnosis of bacterial infection, resulting in delayed immunomodulatory therapy [4]. Laboratory findings such as elevated inflammatory markers, hypoalbuminemia, hyponatremia, anemia, and thrombocytosis can provide important diagnostic clues in such cases [3].

Kawasaki disease shock syndrome (KDSS) is rare but also a serious complication of Kawasaki disease, characterized by hypotension or clinical signs of poor perfusion requiring aggressive fluid resuscitation or vasoactive support [5]. KDSS is associated with an exaggerated inflammatory response, increased capillary leak, and myocardial dysfunction and has been reported in approximately 2%-7% of children diagnosed with KD [5,6]. Children with KDSS, when compared with those with classic KD, have a substantially higher risk of coronary artery abnormalities and an increased incidence of left ventricular systolic dysfunction [6,7]. Clinically, KDSS frequently mimics septic shock, presenting with persistent fever, hypotension, markedly elevated inflammatory markers, and multiorgan involvement, which often leads to diagnostic delay [6]. Distinguishing KDSS from severe infection is critical, as delayed initiation of intravenous immunoglobulin (IVIG) therapy has been associated with poorer cardiac outcomes [7]. We report a case of incomplete Kawasaki disease complicated by KDSS to illustrate the diagnostic challenges, evolving clinical features, and multidisciplinary management required to achieve a favorable outcome.

## Case presentation

**Figure 1:**
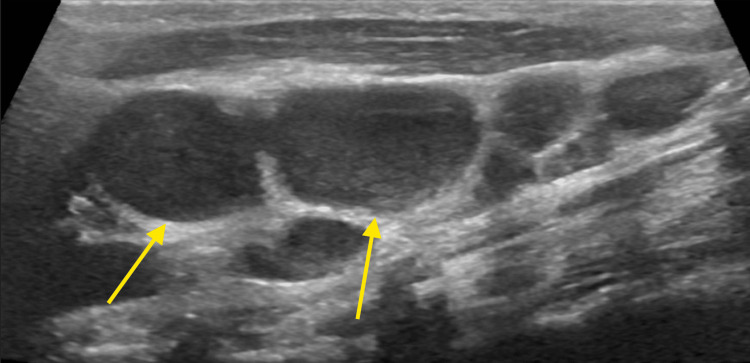
Neck ultrasonography showing right-sided lymphadenopathy (arrows)

An eight-year-old boy, previously healthy and unvaccinated, presented with a two-day history of high-grade fever, left-sided neck pain, and swelling. He had arrived in the United Arab Emirates from Chad one week before the presentation. Examination was unremarkable apart from left-sided cervical lymph node enlargement. Initial evaluation revealed markedly elevated inflammatory markers, as shown in Table 1. A respiratory viral panel was positive for enterovirus/rhinovirus. Blood and urine cultures, urine analysis, tests for malaria, and a group A Streptococcus swab were negative. Ultrasound of the neck showed multiple small, oval-shaped left-sided cervical lymph nodes, with the largest measuring 4.8 mm in short axis, and with no evidence of abscess formation or fluid collections (Figure 1). He was admitted with a working diagnosis of cervical lymphadenitis and started on intravenous clindamycin (13.3 mg/kg three times daily).

**Table 1:** Laboratory test results. WBC: white blood cells, Hb: hemoglobin, PLT: platelets, CRP: C-reactive protein, PCT: procalcitonin, pro-BNP: pro-B-type natriuretic peptide.

	On admission	Day 3 of admission	Day 5 of admission	Day 7 of admission	Day 9 of admission	Follow-up at 2 weeks	Normal range
WBC (10^3/uL)	10.1	6	12.2	10.5	8.8	9.7	5-13
Hb (g/dL)	13.2	12.1	10.1	9.6	10.8	13.3	11.5-15.5
PLT ((10^3/uL))	275	193	202	378	526	390	170-450
CRP (mg/L)	107	205	333	-	61.4	<0.6	0.4-1.3
PCT (ng/mL)	3.4	3.7	22.5	2.8	-	-	<0.05
Sodium (mmol/L)	131	-	-	133	134	-	136-143
Albumin (g/dL)	4.1	-	2.3	2.5	2.9	-	4.2-5.1
Ferritin (ng/mL)	-	1283	1113	-	910	131	14-124
pro-BNP (pg/mL)	-	-	9422	-	-	-	<125

On the third day of hospital admission (day 5 of fever), despite antimicrobial therapy, he continued spiking a fever with increased frequency. Due to a lack of clinical improvement, IV ceftriaxone (75 mg/kg once daily) was added to his antibiotic regimen. At the same time, incomplete Kawasaki disease was considered, given his worsening inflammatory markers and fever pattern; hence, he received IVIG 2 g/kg and was started on aspirin. 

**Figure 2:**
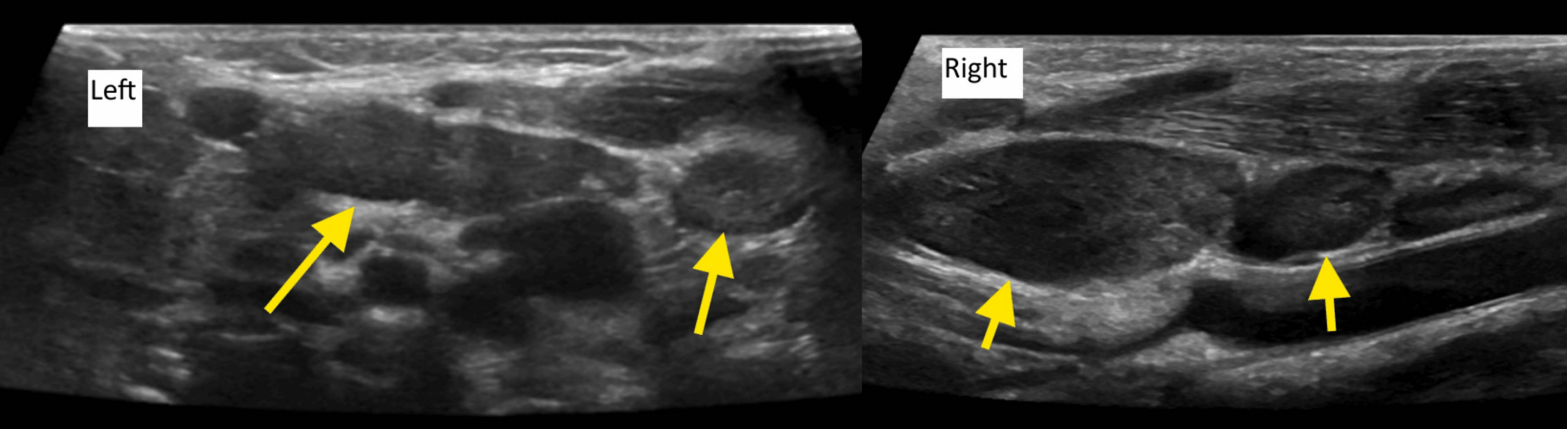
Neck ultrasonography showing left-sided (Left) and right-sided (Right) lymphadenopathy (arrows)

On the following day (admission day 4), the patient developed bilateral non-purulent conjunctivitis, strawberry tongue, and generalized erythematous maculopapular rash, making the clinical picture more consistent with Kawasaki disease. He also complained of neck pain on flexion and rotation, and neck stiffness was also noted. Repeat neck ultrasonography showed bilateral acute cervical lymphadenopathy, without fluid collection, as seen in Figure 2. The neck stiffness was attributed to IVIG-associated aseptic meningitis. Transthoracic echocardiography at that time demonstrated normal intracardiac anatomy and function with normal coronary artery dimensions (left main coronary artery (LMCA) 2.7 mm, Z-score 0.53; left anterior descending artery (LAD) 2.3 mm, Z-score 1.1; and right coronary artery (RCA) 2.3 mm, Z-score 0.94, with no aneurysm).

Overnight, he acutely deteriorated with desaturation, tachypnea, tachycardia, hypotension, and persistent fever (peripheral oxygen saturation (SpO₂) 85%, heart rate 150 bpm, respiratory rate 44/min, blood pressure 75/53 mmHg). Capillary refill time remained under two seconds with strong peripheral pulses. Oxygen saturation picked up with supplementation via face mask; two 10 mL/kg normal saline boluses were administered, which improved blood pressure only transiently.

Given refractory hypotension and a picture of septic shock, the patient was transferred to the pediatric intensive care unit for inotropic support with norepinephrine. Further evaluation showed hypoalbuminemia, elevated pro-B-type natriuretic peptide, D-dimer, prolonged partial thromboplastin time, and rising inflammatory markers, with negative repeat cultures and malaria testing. The infectious disease team advised escalating the antibiotic regimen to vancomycin, meropenem, and doxycycline (which were given for 48 hours until labs came back negative). Broad infectious investigations, including dengue, arboviral, and rickettsial serologies, were sent as summarized in Table 2. Nonsteroidal anti-inflammatory drugs (NSAIDs) - including aspirin - were temporarily withheld pending exclusion of hemorrhagic infections.

**Table 2:** Infectious diseases work-up. enceph: encephalitis, PCR: polymerase chain reaction, IgG: immunoglobulin G, HIV: human immunodeficiency virus, RNA: ribonucleic acid, IgM: Immunoglobulin M, Ag: antigen, Ab: antibody, EBV: Epstein-Barr virus, DNA: deoxyribonucleic acid.

Lab test	Result
Malaria parasite	Negative
Dengue Virus IgG	Positive
Dengue Virus IgM	Negative
Dengue Fever Virus RNA PCR	Negative
Chikungunya virus IgG	Negative
Chikungunya virus IgM	Negative
GI panel (multiplex PCR)	Negative
Brucella IgG	Negative
Brucella IgM	Negative
HIV 1 & 2 Ag & Ab	Non-reactive
Borrelia IgG	Positive
Borrelia IgM	Negative
Rickettsia IgG	Positive
Rickettsia IgM	Negative
Typhus IgG	Negative
Typhus IgM	Negative
Arbo Virus IgG (California enceph, East Equine enceph, st louis enceph, West Equine enceph)	Negative
Arbo Virus IgM (california enceph, east equine enceph, st louis enceph, West Equine enceph)	Negative
Measles Virus RNA PCR-Urine	Negative
Measles Virus IgM	Negative
Measles Virus IgG	Negative
Rubella IgM	Non-reactive
Rubella IgG	Non-reactive
Throat culture	Negative
Urine culture	Negative
blood culture	Negative
Cytomegalovirus IgG	Positive
Cytomegalovirus IgM	Negative
EBV DNA PCR	Negative
Respiratory Screening Panel PCR	Rhino-entero virus positive

On day 5 of admission, repeat echocardiography showed mild cardiac dysfunction with an ejection fraction of 43%, dilated LMCA (3.6 mm, Z-score 2.9) and borderline dilated LAD (2.75 mm, Z-score 2.4), normal RCA (2.7 mm, Z-score 1.39), no aneurysm. Rheumatology opinion favored a diagnosis of Kawasaki disease complicated by Kawasaki shock syndrome over septic shock, given the presence of mucocutaneous features, persistent fever, hypoalbuminemia, hyponatremia, escalating inflammatory markers, and cardiovascular findings. A second dose of IVIG (2 g/kg) was administered, and he was started on intravenous methylprednisolone 1 mg/kg/dose twice daily. His fever settled, and marked clinical improvement was noted 24 hours after starting the steroids, and he was transferred back to the general pediatric ward.

**Figure 3:**
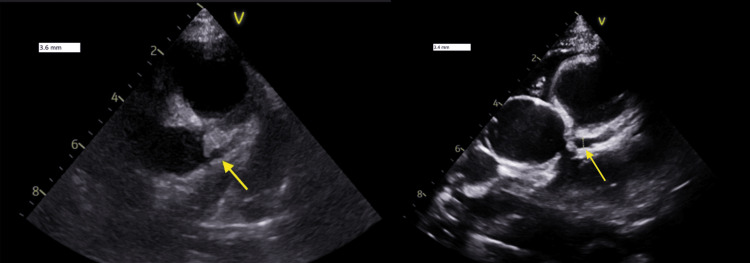
Day 5 echocardiography showing LMCA dilation (left); day 7 echocardiography showing improvement in LMCA dilation (right). Measurements were taken at end-diastole. LMCA: left main coronary artery

Over the following days, he remained hemodynamically stable, afebrile, and aspirin was switched to low dose. Repeat echocardiography on day 7 of admission showed recovered ventricular function, ejection fraction of 61%, improvement in the dilated left main (3.4 mm, Z-score 1.9), right coronary artery remained within normal (2.8 mm, Z-score 1.9) with normal LAD (2.4 mm, Z-score1) (Figure 3). Inflammatory markers were trending down, and after receiving methylprednisolone for 3 days, it was switched to oral prednisolone 2 mg/kg once daily. He remained clinically stable, and on day 9, he was discharged on aspirin and prednisolone.

Two weeks following discharge, he was seen in the clinic doing well, afebrile, and inflammatory markers had normalized; C-reactive protein was the main marker for follow-up, pro-B-type natriuretic peptide was not repeated as the cardiac function had improved, and other markers were trending down. Given that, he was advised aspirin (5 mg/kg once daily) for a total of 6 weeks and to taper off prednisolone.

## Discussion

This case illustrates the diagnostic complexity of incomplete Kawasaki disease when the initial presentation is dominated by focal inflammatory findings. Unilateral cervical lymphadenopathy with neck pain and fever frequently prompts evaluation for infectious etiologies, including bacterial lymphadenitis and deep neck space infections such as retropharyngeal or parapharyngeal abscesses. Several reports have described children with KD undergoing extensive antimicrobial therapy and even surgical evaluation before the inflammatory nature of the illness was recognized [8,9]. In such cases, lack of abscess formation on imaging, persistent fever despite appropriate antibiotics, and progressively rising inflammatory markers should raise suspicion for KD rather than isolated infection [8,9].

The differential diagnosis in these cases is broad and includes bacterial sepsis, deep neck infections, viral illness, and secondary hyperinflammatory syndromes. Deep neck infections are a particularly important mimic, as cervical lymphadenitis-dominant KD may present with neck stiffness, odynophagia, and imaging findings suggestive of inflammatory lymphadenopathy without a drainable collection. Recognition of this pattern is critical, as delayed diagnosis of KD is associated with increased coronary artery involvement [8,9].

The transition from persistent inflammation to hemodynamic instability in this patient marked a critical shift in diagnostic reasoning. Rather than representing isolated septic shock, the combination of escalating inflammatory markers, myocardial dysfunction, and evolving mucocutaneous features supported a unifying inflammatory vasculitic process. In this context, early echocardiographic reassessment and measurement of cardiac biomarkers played a key role in confirming disease severity and guiding escalation of treatment [6,7]. Although severe infection was initially strongly suspected, the clinical course argued against an infectious etiology. The absence of improvement despite broad-spectrum antibiotics, persistently negative blood cultures and serologies, and the progressive emergence of features consistent with Kawasaki disease supported an alternative diagnosis. The rapid and sustained response to IVIG and corticosteroid therapy further reinforced the likelihood of an inflammatory vasculitic process rather than isolated infection.

Kawasaki disease shock syndrome is frequently confused with septic shock due to overlapping clinical features, including fever, hypotension, elevated inflammatory markers, and multiorgan involvement. This overlap often leads to delayed immunomodulatory therapy. Importantly, children with KDSS have higher rates of resistance to initial IVIG therapy compared with those with classic KD [6,7]. Management, therefore, requires a dual approach incorporating aggressive supportive care and timely immunomodulation. Supportive management includes judicious fluid resuscitation, early vasoactive support due to the high prevalence of myocardial dysfunction, respiratory support when indicated, and close hemodynamic monitoring in an intensive care setting [6].

Intravenous immunoglobulin remains first-line therapy; however, adjunctive immunomodulatory agents are frequently required in severe or IVIG-resistant disease. Systemic corticosteroids are commonly used in high-risk presentations, and biologic therapies such as tumor necrosis factor-α inhibitors and interleukin-1 blockade have shown benefit in refractory KD [10]. Awareness of these therapeutic options is essential, as delayed escalation of immunomodulatory therapy has been associated with increased cardiac morbidity.

## Conclusions

This case highlights the diagnostic complexity of incomplete Kawasaki disease presenting with cervical lymphadenitis and subsequent progression to Kawasaki shock syndrome. Clinicians should maintain a high level of suspicion for Kawasaki disease in children with persistent fever and localized inflammatory findings who do not improve with antimicrobial therapy, particularly when imaging fails to identify a drainable collection. Early recognition of KDSS, prompt supportive care, and timely escalation of immunomodulatory therapy are critical to preventing cardiac complications and improving outcomes.
